# The impact of fear of recurrence and progression on mental health after spontaneous coronary artery dissection: the role of illness uncertainty

**DOI:** 10.1093/eurjcn/zvag090

**Published:** 2026-03-25

**Authors:** Sarah T Clarke, Barbara M Murphy, Michael R Le Grande, Michelle C Rogerson, Jason C Kovacic, Stephanie Hesselson, Siiri E Iismaa, Robert M Graham, Robert Hester, Alun C Jackson

**Affiliations:** Australian Centre for Heart Health, The Royal Melbourne Hospital, PO Box 2137, Melbourne, VIC 3050, Australia; Melbourne School of Psychological Sciences, University of Melbourne, Parkville, VIC 3010, Australia; Australian Centre for Heart Health, The Royal Melbourne Hospital, PO Box 2137, Melbourne, VIC 3050, Australia; Melbourne School of Psychological Sciences, University of Melbourne, Parkville, VIC 3010, Australia; Australian Centre for Heart Health, The Royal Melbourne Hospital, PO Box 2137, Melbourne, VIC 3050, Australia; Melbourne School of Psychological Sciences, University of Melbourne, Parkville, VIC 3010, Australia; Australian Centre for Heart Health, The Royal Melbourne Hospital, PO Box 2137, Melbourne, VIC 3050, Australia; Victor Chang Cardiac Research Institute, PO Box 699, Darlinghurst, NSW 2010, Australia; School of Clinical Medicine, St Vincent’s Hospital Healthcare Clinical Campus, Faculty of Medicine and Health, University of NSW, Level 5, deLacy Building, Victoria Street, Darlinghurst, NSW 2010, Australia; Cardiovascular Research Institute, Icahn School of Medicine at Mount Sinai, One Gustave L. Levy Place, New York, NY 10029,USA; Victor Chang Cardiac Research Institute, PO Box 699, Darlinghurst, NSW 2010, Australia; Victor Chang Cardiac Research Institute, PO Box 699, Darlinghurst, NSW 2010, Australia; School of Clinical Medicine, St Vincent’s Hospital Healthcare Clinical Campus, Faculty of Medicine and Health, University of NSW, Level 5, deLacy Building, Victoria Street, Darlinghurst, NSW 2010, Australia; Victor Chang Cardiac Research Institute, PO Box 699, Darlinghurst, NSW 2010, Australia; School of Clinical Medicine, St Vincent’s Hospital Healthcare Clinical Campus, Faculty of Medicine and Health, University of NSW, Level 5, deLacy Building, Victoria Street, Darlinghurst, NSW 2010, Australia; Melbourne School of Psychological Sciences, University of Melbourne, Parkville, VIC 3010, Australia; Australian Centre for Heart Health, The Royal Melbourne Hospital, PO Box 2137, Melbourne, VIC 3050, Australia; Melbourne School of Psychological Sciences, University of Melbourne, Parkville, VIC 3010, Australia; Centre on Behavioral Health, University of Hong Kong, CJT-706, 7/F., The Jockey Club Tower, The Centennial Campus, Pokfulam, Hong Kong

**Keywords:** Anxiety, Cardiac distress, Depression, Fear of recurrence and progression, Psychocardiology, SCAD

## Abstract

**Aims:**

Spontaneous coronary artery dissection (SCAD) is a cause of acute myocardial infarction that is marked by a particularly challenging psychosocial recovery period. Survivors commonly experience fear of recurrent events and uncertainty around their condition. These psychosocial challenges may contribute to anxiety, depression, and cardiac distress. This study investigated the relationship between fear of recurrence and progression (FoRP), uncertainty, and psychological outcomes, specifically whether illness uncertainty moderates the relationship between FoRP and mental health outcomes in SCAD survivors.

**Methods and results:**

Participants were recruited from the **V**ictor Chang Cardiac Research Institute **A**rteriopathy and **S**CAD **C**ohort (VASC). Two hundred and seventy survivors completed an online questionnaire measuring FoRP, illness uncertainty, anxiety, depression, and cardiac distress. Moderation analyses were undertaken to investigate the relationship between these variables. FoRP directly predicted all three mental health variables. Illness uncertainty moderated this relationship in the case of depression and cardiac distress.

**Conclusion:**

The findings indicate specific intervention targets to meet the support needs of survivors. While fear of recurrent events is common following SCAD, managing illness uncertainty may be a means to reduce the impacts of this fear on survivor's mental health.

## Graphical Abstract

**Graphical Abstract:**
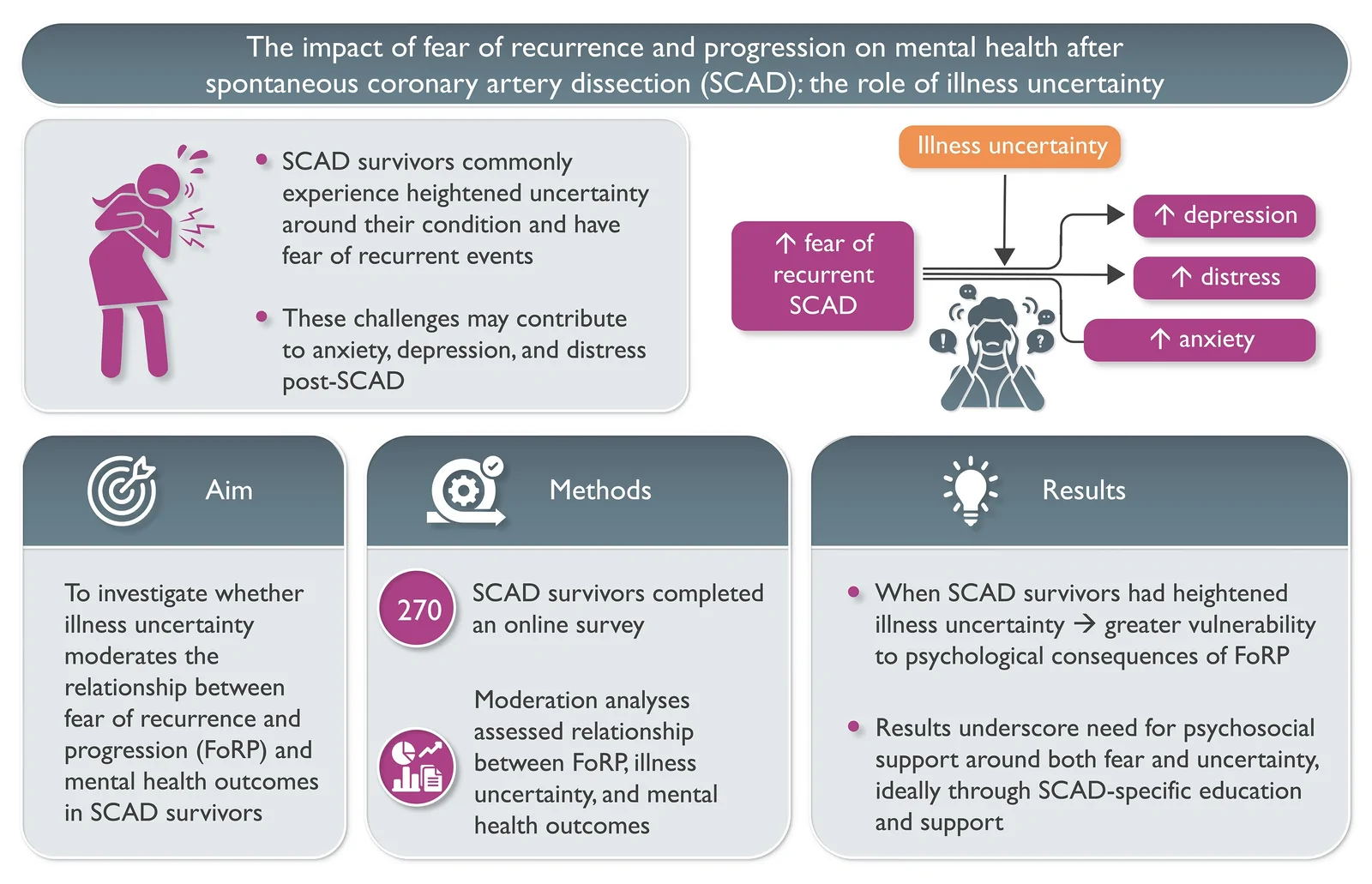
FoRP = fear of recurrence and progression; SCAD = Spontaneous Coronary Artery Dissection

Novelty

- This study is the first to demonstrate that illness uncertainty intensifies the psychological burden of fear of recurrence and progression (FoRP) among Spontaneous Coronary Artery Dissection (SCAD) survivors, particularly in relation to depression and cardiac distress.
- It highlights illness uncertainty as a key mechanism through which FoRP translates into poorer mental health, pointing to a novel target for psychosocial interventions.
- The findings challenge the adequacy of existing FoRP measurement tools in SCAD and cardiac populations more broadly, suggesting that current scales may underestimate its true impact and underscore the need for condition-specific assessment tools.

## Introduction

There has been growing research interest in the psychosocial impacts of spontaneous coronary artery dissection (SCAD) alongside awareness of the significant and persistent distress experienced by survivors.^1^ SCAD is an increasingly acknowledged cause of acute myocardial infarction (AMI) whereby an intramural haematoma results in coronary artery occlusion.^2–4^ The heightened distress may relate to the somewhat atypical profile of SCAD-induced AMI, with SCAD typically occurring in mid-aged women who present with few conventional atherosclerotic risk factors, and is marked by relatively high rates of recurrence.^5,6^ Indeed, around 35% of survivors report SCAD-induced post-traumatic stress disorder (PTSD).^7^ Anxiety and depression may be even more common, with symptoms of at least mild anxiety in 41–49% of survivors and mild depression in 32–49%.^8,9^ More general psychosocial impacts, such as shock, fear, confusion, and uncertainty, are experienced in up to 90% of survivors.^10,11^

As part of this broader psychosocial impact profile, it is common for SCAD survivors to report a fear of recurrence.^12–15^ Some describe this fear as all-consuming and the most challenging component of recovery.^13^ Others describe this fear as a trigger for symptoms of anxiety, depression, and post-traumatic stress disorder after SCAD, which prompts them to seek mental health support.^12^ In chronic illness research, the term ‘fear of recurrence and progression’ (FoRP) is used to define the fear that a disease will progress or recur, with all its consequences.^16–18^ SCAD research suggests that FoRP may be experienced by almost all survivors in the period immediately following their event. Specifically, in the six-months after SCAD, 98% of survivors report worrying about having another SCAD, with 81% of survivors experiencing this to a moderate or large degree.^10,11^ Indeed, concerns about recurrence in SCAD are over double the rates found in other cardiac populations.^18^ To understand why FoRP may be particularly high following SCAD, it is important to consider the illness context.

SCAD survivors are particularly challenged by the uncertainty surrounding their condition.^12,13^ Uncertainty in illness is a construct used to describe the inability to form meaning in one's illness, due to the illness experience being vague, ill-defined, unpredictable, and/or ambiguous.^19,20^ In SCAD, uncertainty is heightened by the lack of clear information and guidelines regarding its causes, management, and treatment.^1,13,14,21,22^ Indeed, both qualitative and quantitative studies have demonstrated that uncertainty in managing one's condition and resuming one's previous lifestyle without triggering a SCAD-recurrence is central to post-SCAD distress.^10,13^ It is therefore likely that, under conditions of uncertainty, SCAD survivors will report heightened FoRP.^13,14,21^ In chronic illness research more broadly, uncertainty has been implicated in poorer psychological adjustment to illness.^23^ In SCAD, both FoRP and illness uncertainty are associated with anxiety and depression.^10^ However, the nature of the relationship between FoRP and illness uncertainty, and how this relationship may impact mental health, has not been directly investigated, neither in SCAD nor in other cardiac patient groups.

It is possible that the high rates of FoRP reflect the high levels of illness uncertainty experienced by SCAD survivors. In cardiology and other chronic illness research, FoRP and illness uncertainty appear to be intrinsically interrelated.^24–29^ For example, anxiety about uncertain physical sensations and fear of recurrence were identified as the main reasons for re-admission in patients following percutaneous coronary intervention (PCI).^30^ Similarly, in breast cancer patients, illness uncertainty has been shown to predict fear of cancer recurrence.^31,32^ While these studies indicate that illness uncertainty and FoRP are related, they do not indicate how this relationship contributes to ongoing mental health outcomes, such as anxiety, depression, and distress.

Psychosocial support is important for SCAD recovery.^3,21,33^ Survivors request psychological support that is specifically tailored to their unique experiences and concerns, particularly involving guidance around living with the possibility of SCAD recurrence and managing anxiety produced by fear and uncertainty.^12–15,22,33,34^ Understanding the relationship between uncertainty, FoRP, and mental health can inform theory-driven interventions that specifically meet the support needs of SCAD survivors. As such, the current study aimed to investigate whether illness uncertainty moderates the relationship between FoRP and mental health outcomes such as anxiety, depression, and cardiac distress in SCAD survivors.

## Method

The current study involved a secondary analysis of data from a larger study on the psychosocial impacts of SCAD.^10^ The project was approved by the Human Research Ethics Committee (HREC) of St Vincent's Hospital Sydney, Australia (HREC/16/SVH/338). Details of the study sample and data collection procedures are presented in full elsewhere^10^ and briefly here.

### Procedure

Data were collected between 10 February and 6 May 2023. Participants were recruited from the **V**ictor Chang Cardiac Research Institute **A**rteriopathy and **S**CAD **C**ohort (VASC)^35^ database of SCAD survivors assembled by the Victor Chang Cardiac Research Institute. All potential participants (*n* = 433) were sent a unique link to the study Participant Information and Consent Form (PICF). A total of 310 consented to participate and were given access to the one-off online questionnaire. Participants accessed the questionnaire via Research Electronic Data Capture (REDCap).^36^ All information was self-reported, and no identifying information was collected. Responses were stored on a password-protected secure server accessible only to authorized members of the research team. Due to the sensitive nature of the information being collected, the PICF provided contact details for participants to request an independent, cost-free counselling session if questionnaire completion caused distress.

### Measures

Socio-demographic information was collected to characterize participants.^10^ The following measures were included in the online questionnaire:

#### Fear of progression questionnaire- short form (FoP-Q-SF)^37^

The FoP-Q-SF is a 12-item measure adapted from the full 43-item measure.^38^ Items measure FoRP, for example, ′*I become anxious if I think my illness may progress*’. Items are scored on a 5-point scale ranging from 1 (never) to 5 (very often), with an overall score range between 12–60. Higher values indicate higher fear. The measure was developed for use in cancer, diabetes, and rheumatic diseases,^38^ and has since been validated in a range of illness populations.^37,39–42^ Although used previously with cardiac patients,^43–45^ the FoP-Q-SF has not been validated in a cardiac context.

##### Psychometric assessment of the FoP-Q-SF

In the current study, the convergent and divergent validity of the FoP-Q-SF were evaluated by assessing the correlations between FoP-Q-SF and related or unrelated measures, respectively (see Table S1). Measures used for validation purposes included the 7-item Generalized Anxiety Disorder scale (GAD-7),^46^ 9-item Patient Health Questionnaire (PHQ-9), the 4-item University of California Los Angeles Loneliness Scale (UCLA-LS), ^47^ the 21-item Post-Traumatic Growth Inventory (PTGI),^48^ and the Mental Component Summary (MCS-12) and Physical Component Summary (PCS-12) from the 12-item Short Form Health Survey (SF-12).^49^ Pre-defined hypotheses for expected correlation values were set, based on previous validation studies of the FoP-Q-SF in other clinical samples.^37,39–42^ Construct validity was considered to be met as over 75% of the hypotheses were met.^50^ The Cronbach's alpha in this study was 0.87, supporting the instruments’ internal consistency. The most commonly endorsed item was ‘*when I am anxious, I have physical symptoms, e.g. rapid heartbeat, stomach-ache, nervousness’.* This item has been criticized as lacking content validity, as it assesses general anxiety rather than FoRP.^51^ Similarly, this item is likely to be particularly salient to cardiac patients compared to other illness populations due to the explicit reference to rapid heartbeat. When this item was removed from the FoP-Q-SF, construct validity was retained (*Table S1*), and Cronbach's alpha remained unchanged. The least commonly endorsed item was ‘*It disturbs me that I may have to rely on strangers for activities of daily living’.* The mean and standard deviation of each item of the FoP-Q-SF is presented in *Table S2*.

#### Mishel's uncertainty in illness scale community version (MUIS-C)^52^

The MUIS-C is a 23-item scale measuring uncertainty in illness in people in the community, for example, ′*I am unsure if my illness is getting better or worse*’. This measure was adapted from the original MUIS with the removal of items referring to treatment in an acute hospital setting. This scale has been established as valid and reliable in a range of cardiac patient poulations.^52,53^ Items are scored from 1 (strongly disagree) to 5 (strongly agree). Six of the 23 items are reverse-coded, and then all items are summed with total scores ranging from 23 to 115. Higher scores indicate higher uncertainty.

#### Mental health outcomes

Anxiety was measured using the GAD-7.^46^ Depression was measured using PHQ-9.^54^ Scores ≥ 10 on the GAD-7 and the PHQ-9 were used as cut-off scores for anxiety and depression.^46,54^ The GAD-7 and PHQ-9 are the tools recommended by the European Society of Cardiology (ESC) for assessment of anxiety and depression in cardiac patients.^55^ Cardiac distress was measured using the 12-item Cardiac Distress Inventory—Short Form (CDI-SF).^56^ A score ≥ 13 on the CDI-SF was used as a cut-off score for clinically significant cardiac distress.

### Data analysis

Analyses were completed using jamovi version 2.3. 28.0.^57^ Data cleaning was undertaken to remove repeat entries and participants who did not have a confirmed SCAD, as described in full previously.^10^ The minimum data set included completion of the mental health outcomes, FoP-Q-SF, and MUIS-C. Moderation analyses were conducted using FoRP as the predictor, illness uncertainty as the moderator, and mental health outcomes (anxiety, depression, and cardiac distress) as the dependent variables. Multicollinearity was determined through a variance inflation factor (VIF) greater than five. Following observation of the highest-ranking item on the FoP-Q-SF in the sample being of questionable content validity (see measures), and potentially reflecting general anxiety, we hypothesized that this item may inflate the direct effect with the GAD-7 and overpower any interaction effects of illness uncertainty. To assess this hypothesis, the moderation analyses were re-run post-hoc using a modified FoP-Q-SF with this item removed.

## Results

### Participant characteristics

Of the 310 eligible participants, 270 completed the minimum data set and were therefore included in the analyses. Ages ranged from 34 to 77 years (*M* = 55.69 years, *SD* = 8.81). Other participant characteristics are shown in *Table 1*. The means and standard deviation of the FoP-Q-SF, MUIS-C, and mental health outcomes are shown in *Table 2*.

**Table 1:** Clinical characteristics (*n* = 270)

Sociodemographic characteristics		*n (%)*
Female gender		258 (96%)
Born in Australia		211 (78%)
*Education level:*	Secondary	37 (14%)
	Mid-level (trade)	60 (22%)
	University	173 (64%)
Partnered		213 (79%)
In paid employment		222 (82%)
Private health insurance		208 (77%)
**Medical characteristics**		
*Time since last SCAD:*	Within past year	34 (13%)
	Between 1–2 years	41 (15%)
	Between 2–3 years	35 (13%)
	Between 3–4 years	34 (13%)
	Between 4–5 years	28 (10%)
	5 years or more	98 (36%)
*Total number of SCADs:*	One	211 (78%)
	Two	38 (14%)
	Three	14 (5%)
	Four	4 (1%)
	Five	3 (1%)
*SCAD treatments:*	Medication	224 (83%)
	Percutaneous coronary intervention	40 (15%)
	Coronary artery bypass surgery	4 (1%)
	Implantable cardioverter defibrillator	3 (1%)
*SCAD specific risk factors:*	Migraine	90 (33%)
	Fibromuscular dysplasia	52 (19%)
	Inflammatory disorder	20 (7%)
	Connective tissue disorder	5 (2%)
*Traditional risk factors:*	High blood pressure	77 (29%)
	High cholesterol	35 (13%)
	Obesity	27 (10%)
	Diabetes/Gestational diabetes	10 (4%)
	Cigarette smoking	4 (1%)
**Psychosocial characteristics**		
Anxious (GAD-7 ≥ 10)		54 (20%)
Depressed (PHQ-9 ≥ 10)		56 (21%)
Distressed (CDI-SF ≥ 13)		41 (15%)

SCAD, Spontaneous Coronary Artery Dissection; GAD-7, Generalized Anxiety Disorder 7; PHQ-9, Patient Health Questionnaire 9; CDI-SF, Cardiac Distress Inventory Short Form

**Table 2:** Clinical descriptive data

	Mean ± SD	Min	Max
FoP-Q-SF	27.68 ± 8.99	12	52
MUIS-C	54.67 ± 14.97	23	99
GAD-7	5.45 ± 4.96	0	21
PHQ-9	6.17 ± 5.69	0	27
CDI-SF	5.71 ± 6.63	0	33

FoP-Q-SF, Fear of Progression Questionnaire Short Form; MUIS-C, Mishel Uncertainty in Illess Scale—Community; GAD-7, Generalized Anxiety Disorder 7; PHQ-9, Patient Health Questionnaire 9; CDI-SF, Cardiac Distress Inventory Short Form

### Moderation analyses

**Figure 1:**
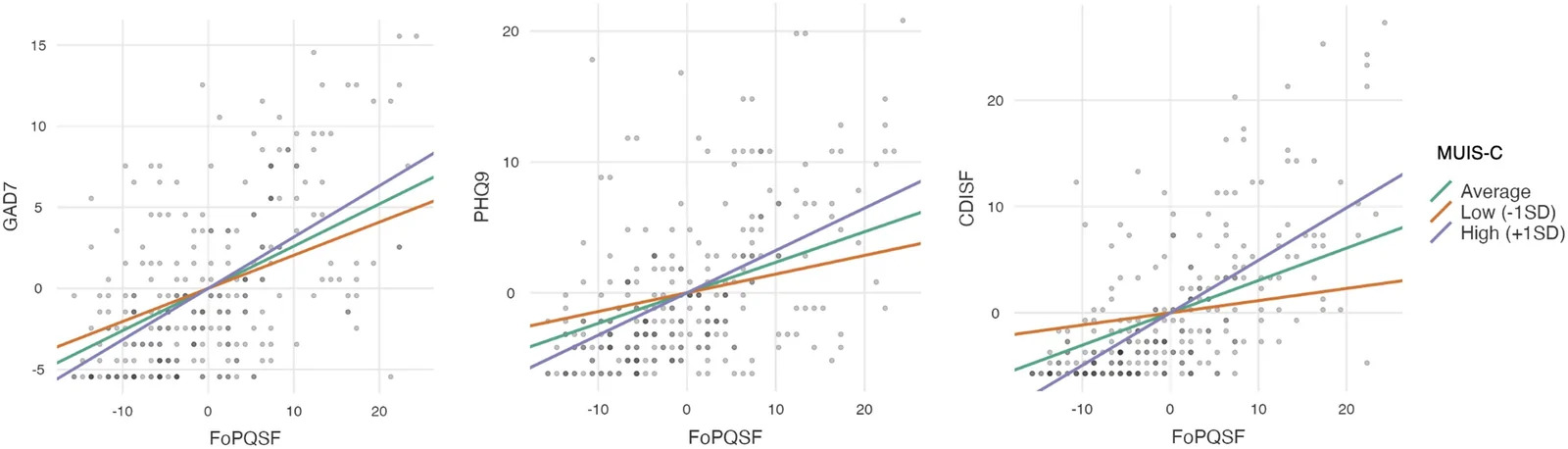
Conditional effect of FoRP on mental health outcomes based on level of illness uncertainty. *Panel 1:* Conditional effect of FoRP on anxiety based on level of illness uncertainty, *Panel 2:* Conditional effect of FoRP on depression based on level of illness uncertainty *Panel 3:* Conditional effect of FoRP on cardiac distress based on level of illness uncertainty. FoP-Q-SF = fear of progression questionnaire short form, MUIS-C = Mishel Uncertainty in Illness Scale—Community, GAD-7 = Generalized Anxiety Disorder 7, PHQ-9 = Patient Health Questionnaire, CDI-SF = Cardiac Distress Inventory—Short Form, SD = standard deviation.

The main and interaction effects of the moderation analyses are shown in *Table 3*. The VIF = 1.66, indicating no significant multicollinearity between FoRP and illness uncertainty. A significant interaction effect was observed on the PHQ-9 and CDI-SF, but not for GAD-7, demonstrating that illness uncertainty moderated the relationship between FoRP with depression and cardiac distress, but not anxiety. Examination of the conditional effects showed the impact of FoRP on depression was stronger for those with higher illness uncertainty [MUIS-C + 1SD: *β* = 0.32, *CI* (0.21, 0.43), *Z* = 5.93, *P* < 0.001] than those with lower illness uncertainty [MUIS-C −1SD: *β* = 0.14, *CI* (0.03, 0.25), *Z* = 2.55, *P* = 0.01], as demonstrated in the *Figure 1* panel 1. The same relationship was revealed between FoRP and cardiac distress (MUIS-C + 1SD: *β* = 0.49, *CI* [0.38, 0.61], *Z* = 8.42, *P* < 0.001; MUIS-C −1SD: *β* = 0.11, *CI* [0.01, 0.21], *Z* = 2.16, *P* = 0.03), as demonstrated in *Figure 1,* panel 2. While a similar pattern was evident for the relationship between anxiety and FoRP scores (MUIS-C + 1SD: *β* = 0.32, *CI* [0.22, 0.41], *Z* = 6.30, *P* < 0.001, MUIS-C −1SD: *β* = 0.20, *CI* [0.09, 0.30], *Z* = 3.71, *P* < 0.001), as demonstrated in *Figure 1* panel 3, there was no statistically significant moderation effect (*P* = 0.07).

**Table 3:** Moderation estimates for each dependant variable

	GAD-7				PHQ-9				CDI-SF			
	β (95% CI)	SE	Z	*P*	β (95% CI)	SE	Z	*P*	β (95% CI)	SE	Z	*P*
**FoP-Q-SF**	0.26 (0.17–0.34)	0.04	6.12	< 0.001	0.23 (0.14–0.32)	0.05	5.08	< 0.001	0.30 (0.21–0.40)	0.05	6.66	< 0.001
**MUIS-C**	0.02 (−0.02–0.07)	0.02	0.99	0.32	0.07 (0.01–0.12)	0.03	2.50	0.01	0.11 (0.06–0.17)	0.03	4.38	< 0.001
FoP-Q-SF* MUIS-C	0.00 (0.00–0.01)	0.00	1.80	0.07	0.01 (0.00–0.01)	0.00	2.64	0.01	0.01 (0.01–0.02)	0.00	6.09	< 0.001

FoP-Q-SF, fear of progression questionnaire short form; MUIS-C, Mishel's uncertainty in illness community scale; GAD-7, Generalized Anxiety Disorder 7; PHQ-9, Patient Health Questionnaire; CDI-SF, Cardiac Distress Inventory—Short Form; SE, standard error; CI, confidence interval.

### Post-hoc analyses

Results of the additional moderation analyses with the revised FoP-Q-SF are shown in *Table 4* and in the Supplementary material online, *Figure S1*. As shown, the interaction effect between illness uncertainty and FoRP on anxiety has now reached significance (*P* = 0.04). Examination of the conditional effects also demonstrated a stronger moderation effect for all three outcome variables. In particular, when illness uncertainty was 1SD below the mean, there was no longer a significant association between cardiac distress and FoRP, underscoring the importance of illness uncertainty as the moderator: (GAD7: MUIS-C + 1SD: *β* = 0.32, *CI* [0.21, 0.41], *Z* = 6.03, *P* < 0.001; MUIS-C −1SD: *β* = 0.19, *CI* [0.06, 0.31], *Z* = 3.04, *P*—0.002; PHQ-9: MUIS-C + 1SD: *β* = 0.33, *CI* [0.20, 0.44], *Z* = 5.52, *P* < 0.001, MUIS-C −1SD: *β* = 0.12, *CI* [−0.01, 0.24], *Z* = 2.03, *P* = 0.04; CDI-SF: MUIS-C + 1SD:: *β* = 0.51, *CI* [0.39, 0.63], *Z* = 8.27, *P* < 0.001; MUIS-C −1SD: *β* = 0.09, *CI* [−0.02, 0.20], *Z* = 1.59, *P* = 0.11).

**Table 4:** Moderation estimates for each dependant variable with modified FoP-Q-SF

	GAD-7				PHQ-9				CDI-SF			
	β (95% CI)	SE	Z	*P*	β (95% CI)	SE	Z	*P*	β (95% CI)	SE	Z	*P*
**FoP-Q-SFr**	0.25 (0.16–0.34)	0.05	5.26	< 0.001	0.23 (0.13–0.32)	0.05	4.73	< 0.001	0.30 (0.21–0.39)	0.05	6.58	< 0.001
**MUIS-C**	0.03 (−0.02–0.08)	0.02	1.36	0.18	0.07 (0.02–0.13)	0.03	2.76	0.006	0.12 (0.07–0.18)	0.03	4.46	< 0.001
FoP-Q-SFr* MUIS-C	0.00 (0.00–0.01)	0.00	2.08	0.04	0.01 (0.00–0.01)	0.00	2.85	0.004	0.01 (0.01–0.02)	0.00	6.46	< 0.001

FoP-Q-SF, fear of progression questionnaire short form; MUIS-C, Mishel's uncertainty in illness community scale; GAD-7, Generalized Anxiety Disorder 7; PHQ-9, Patient Health Questionnaire; CDI-SF, Cardiac Distress Inventory—Short Form; SE, standard error; CI, confidence interval.

## Discussion

This study examined how illness uncertainty influences the relationship between FoRP and mental health outcomes in SCAD survivors. FoRP directly predicted depression, anxiety, and cardiac distress. Illness uncertainty moderated this relationship in the case of depression and cardiac distress, and, for the modified FoRP scale, also anxiety. These results build on the growing understanding of the psychosocial burdens of SCAD, specifically regarding the role of illness uncertainty in the transition to more serious mental health outcomes. Previous studies suggested that SCAD survivors with heightened uncertainty regarding how to best manage their condition also experienced heightened fear of SCAD recurrence.^10,13,14,21^ While both FoRP and illness uncertainty are associated with anxiety and depression,^10^ the combined impact of these two constructs on mental health in SCAD remained unclear. The current findings suggest that when SCAD survivors are in a heightened state of illness uncertainty—whether that be related to a lack of information, unclear medical guidance, or unpredictable triggers—they are more vulnerable to the psychological consequences of FoRP. These results suggest specific intervention targets for meeting the support needs of survivors, specifically surrounding support in living with uncertainty and managing FoRP.

Almost all SCAD survivors experience FoRP,^10,11^ and by addressing illness uncertainty, we may be able to minimize the impact of such fear on survivors’ mental health. From a service-related perspective, illness uncertainty may be ameliorated through the provision of adequate and clear information about SCAD.^24^ This may include, where possible, providing information about SCAD recurrence risk, clarification on triggers, and the formulation of specific recovery plans, including information on what survivors can and cannot do.^13^ While position statements and resources exist to guide practitioners providing this information,^2,5,58^ all were published more than five years ago. There is a clear need for updated management guidelines to better support information delivery. Consideration needs to be given to how best to provide information to SCAD survivors in time-poor healthcare settings.^24^

In addition to information, SCAD survivors can benefit from evidence-based psychosocial treatments. Such interventions can support survivors to adapt to illness uncertainty through acceptance, a critical step within Mishel's model of uncertainty in illness.^19,24^ Acceptance and commitment therapy-based interventions, such as those described in recently published SCAD support programs,^33,59^ may be useful in this context by building tolerance and acceptance towards uncertainty.^13^

Nonetheless, the role of FoRP in the psychological profile of SCAD is not yet clear. The results of this study, as well as FoRP research in SCAD and cardiology more broadly, is limited in its measurement of FoRP using the FoP-Q-SF. It is likely that this measure does not adequately capture the experience of FoRP in SCAD survivors. Notably, the FoP-Q-SF scores reported were lower than those in studies of patients with other cardiac^44,60^ and chronic illness samples.^40,61,62^ This may suggest that the SCAD survivors find the FoP-Q-SF items less salient than other chronic illness cohorts, and even other cardiac presentations, despite SCAD survivors harbouring a uniquely high risk for FoRP.^10,11,17,18^ While concurrent and discriminant validity were investigated, no other FoRP measures were available at the time of data collection with which to assess convergent validity of the FoP-Q-SF. Indeed, several items have questionable face validity, both in measuring the construct of FoRP^51^ and in their relevance in SCAD and cardiac samples more broadly.^18^ Item content clearly had a critical influence on the results, with the post-hoc removal of a single item strengthening the moderating effect of illness uncertainty between FoRP and all three mental health outcome variables, and importantly, resulting in illness uncertainty becoming a significant moderator of anxiety. Similarly, being applicable across a range of chronic illnesses, the FoP-Q-SF omits items that are particularly salient in SCAD and broader cardiac samples, such as the fears of physical activity resulting in recurrence.^13,18^ This may suggest that the FoP-Q-SF has underestimated the true burden of FoRP in this sample. A cardiac-FoRP measure that may more accurately approximate the relationship between these variables in a SCAD sample is currently in development.^63^ Similarly, limiting the MUIS-C is a transdiagnostic measure of illness uncertainty. Incorporating SCAD-specific items would enable a more precise examination of which specific aspects of illness uncertainty in SCAD drive the moderation effect, thereby identifying clearer targets for intervention.

The present study had additional limitations. First, the cross-sectional nature of the data limited the causal and temporal inferences that could be drawn from the results. Second, the measures required for the minimum data set were included towards the end of the larger survey pack, increasing dropout attrition. This resulted in 40 participants being excluded from the complete-case analysis, reducing the sample size and potentially introducing bias. Third, the sample was relatively homogenous with participants mostly being partnered, employed, University-educated Australian-born women, and only 13% of the sample having had their SCAD in the previous year. Consequently, these results may not be representative of all SCAD survivors, particularly those in the acute recovery period directly post-SCAD. Relatedly, the focused scope of this study did not include covariates such as age, sex, pre-existing mental health conditions, or time since-SCAD. Future research should examine how these demographic and clinical factors influence the relationships between illness uncertainty, FoRP, and mental health outcomes. Specifically, longitudinal research in more diverse samples can help more robustly determine how these relationships evolve over time and throughout the recovery process.

## Conclusions

This study contributes to the growing understanding of the psychological impact of SCAD. The present findings suggest that FoRP directly influences anxiety, depression, and cardiac distress, and that these impacts may be more pronounced in those experiencing higher illness uncertainty. These results underscore the need for psychosocial support around both fear and uncertainty, ideally through SCAD-specific education and support. Future research should prioritize the development and validation of cardiac-specific FoRP measures and examine these relationships longitudinally in more diverse SCAD populations to guide tailored, evidence-based care.

## Supplementary Material

zvag090_Supplementary_Data

## Data Availability

The data underlying this article are available in the University of Melbourne Figshare repository at: https://doi.org/10.26188/24654414.v1.
