# Supplementary material for: The impact of fear of recurrence and progression on mental health after spontaneous coronary artery dissection: the role of illness uncertainty
Source: Eur J Cardiovasc Nurs. 2026 Mar 25;25(6):1210–8. doi: 10.1093/eurjcn/zvag090 (PMC13549710; doi:10.1093/eurjcn/zvag090)
Supplement: Supplementary file 1 [file zvag090_supplementary_data.docx]

**Supplementary Materials**

**Table S1. Correlations between FoP-Q-SF and validation measures**

| **Validation scale** | **N** | **Pearson’s R (original FoP-Q-SF)** | **Pearson’s R (modified FoP-Q-SF)** | **P-value** | **Confirmed** |
| --- | --- | --- | --- | --- | --- |
| *Pre-defined hypothesis: moderate to large correlation* | | | | |  |
| GAD-7 | 274 | 0.55 | 0.52 | < 0.001 | Yes |
| PHQ-9 | 274 | 0.52 | 0.50 | < 0.001 | Yes |
| MCS-12 | 274 | -0.47 | -0.45 | < 0.001 | Yes |
| *Pre-defined hypothesis: small or no correlation* | | | | |  |
| PCS-12 | 274 | -0.49 | -0.49 | < 0.001 | No |
| UCLA-LS | 268 | 0.30 | 0.28 | < 0.001 | Yes |
| PTGI | 263 | 0.05 | 0.05 | 0.40* | Yes |

**Legend**: Correlations were interpreted as small if r ≤ 0.3, moderate if r > 0.3 and < 0.5, and large if r ≥ 0.5. N, p-value and hypothesis confirmation values same between original and modified FoP-Q-SF with exception of where marked with * where p-value = 0.41. GAD-7 = FoP-Q-SF = Fear of Progression Questionnaire Short Form; Generalised Anxiety Disorder 7; PHQ-9 = Patient Health Questionnaire 9; MCS-12 = Mental Component Summary from the Short Form 12; PCS-12 = Physical Component Summary from the Short Form; UCLA-LS = University of California Loneliness Scale; PTGI = Post-Traumatic Growth Inventory.

**Table S2. Mean and standard deviation for each item of FoP-Q-SF (N = 274)**

| **FoP-Q-SF item** | **Mean ± SD** |
| --- | --- |
| I become anxious if I think my illness may progress | 2.50 ± 0.96 |
| I am nervous prior to doctor’s appointments or periodic examinations | 2.34 ± 1.23 |
| I am afraid of pain | 2.18 ± 1.01 |
| The thought that I might become less productive at my job disturbs me | 1.97 ± 1.09 |
| When I am anxious, I have physical symptoms, e.g., rapid heartbeat, stomach-ache, nervousness | 2.89 ± 1.16 |
| The possibility of my children developing a SCAD disturbs me | 2.69 ± 1.28 |
| It disturbs me that I may have to rely on strangers for activities of daily living | 1.72 ± 1.05 |
| I am worried that at some point in time, because of my illness I will no longer be able to pursue my hobbies | 2.07 ± 1.15 |
| I am afraid of severe medical treatments in the course of my illness | 2.06 ± 1.13 |
| I worry that my medications could damage my body | 2.49 ± 1.30 |
| I worry about what will become of my family if something should happen to me | 2.78 ± 1.28 |
| The thought that I might not be able to work due to my illness disturbs me | 2.05 ± 1.22 |

**Legend**: FoP-Q-SF = Fear of Progression Questionnaire Short Form

**Supplemental Figure.** Conditional effect of FoRP (using modified FoP-Q-SF) on mental health outcomes based on level of illness uncertainty.

MUIS-C

**Legend:** *Panel 1:* Conditional effect of FoRP (using modified FoP-Q-SF) on anxiety scores based on level of illness uncertainty, *Panel 2:* Conditional effect of FoRP (using modified FoP-Q-SF) on depression scores based on level of illness uncertainty, *Panel 3*: Conditional effect of FoRP (using modified FoP-Q-SF) on cardiac distress scores based on level of illness uncertainty. FoP-Q-SF = fear of progression questionnaire short form, GAD-7 = Generalised Anxiety Disorder 7, PHQ-9 = Patient Health Questionnaire, CDI-SF = Cardiac Distress Inventory – Short Form, SD = standard deviation
